# Beta-Cell Secretory Function in Recent-Onset Type 2 Diabetes Mellitus: A Comparative Study Across Body Mass Index Categories

**DOI:** 10.7759/cureus.92288

**Published:** 2025-09-14

**Authors:** Sarin S M, Balakrishnan Valliyot, Pramod V K

**Affiliations:** 1 Department of Internal Medicine, Government Medical College, Kannur, IND

**Keywords:** abdominal obesity, beta-cell dysfunction, homeostasis model assessment, insulin resistance, lean weight, normal weight, overweight, type 2 diabetes mellitus

## Abstract

Introduction: A section of type 2 diabetes mellitus (T2DM) patients have normal/lean body weight as opposed to classic diabetic patients who are generally overweight. The relative role of insulin resistance and beta-cell secretory defect in lean-/normal-weight T2DM patients as compared to patients of overweight body type needs further inquiry.

Methods: This cross-sectional study, conducted in family health centres across North Kerala, included 120 T2DM patients aged ≥30 years with a disease duration of ≤5 years. Participants were stratified into lean-weight, normal-weight, and overweight groups based on body mass index (BMI), with 40 patients randomly selected per group from an initial cohort of 829 screened patients. Coupled fasting serum glucose and insulin levels were estimated after an eight-hour overnight fasting serum sample. The homeostasis model assessment 2-measure of insulin resistance (HOMA2-IR) and homeostasis model assessment 2-measure of beta-cell secretion (HOMA2-%B) were then calculated using these measures with the help of the HOMA2 calculator. Data analysis was performed using RStudio Version 2023.06.1+524 (Posit Software, Boston, MA, USA).

Results: In the study population, comparison across three body weight categories revealed that waist circumference (WC), hip circumference, fasting insulin, HOMA2-IR, and HOMA2-%B differed significantly among them. Mean HOMA2-%B values were lower in lean-weight (27.38±22.18) and normal-weight (30.0±24.7) patients and highest in the overweight group (46.9±26.3). Similarly, HOMA2-IR was markedly elevated in overweight individuals (1.28±0.65) compared to lean-weight (0.72±0.55) and normal-weight (0.73±0.56) groups. Post hoc Tukey HSD analysis confirmed that only the overweight category patients had a statistically significant difference for these variables from the other two groups. Patients with abdominal obesity, defined by South Asian WC cut-offs, had significantly higher HOMA2-IR and HOMA2-%B values than those without. Regression analysis indicated that a 10% increase in BMI was associated with a 16.04% rise in HOMA2-%B and a 19.34% rise in HOMA2-IR. Female patients show 41.55% higher HOMA2-%B and 31.78% higher HOMA2-IR than males. Each additional year in age of onset of diabetes predicts a 1.71% rise in HOMA2-%B with no significant impact on insulin resistance.

Conclusion: Lean- or normal-weight diabetic patients have lower beta-cell secretion compared to overweight/obese patients. Also, patients with abdominal obesity exhibit higher insulin resistance with better preserved beta-cell function compared to those without. Also, patients with a younger age of onset of diabetes have comparatively lower beta-cell reserve compared to those developing diabetes at an older age.

## Introduction

Type 2 diabetes mellitus (T2DM) is one of the most common lifestyle diseases affecting about 10.5% of the world's adult population. In India, an estimated 74.2 million patients have T2DM by the year 2021, and the overall incidence over a 10-year follow-up stands between 20.2 and 24.5 per person-years [1,2]. Complications secondary to diabetes are the leading causes of blindness, end-stage renal disease, and non-traumatic amputation of extremities. It is also an important risk factor for the rising prevalence of coronary artery disease. Early detection and treatment of T2DM are thus essential in preventing these complications and reducing the long-term morbidity and mortality associated with the disease [3].

Effective management of a diabetic patient needs an in-depth understanding of the pathogenesis of the disease process. The pathophysiological mechanism of T2DM mostly involves insulin resistance accompanied by concurrent beta-cell secretory dysfunction. Classical obese T2DM patients are characterized by significant insulin resistance accompanied by a relative deficiency in beta-cell secretion. Recent investigations indicate that normal-weight/lean-weight persons with T2DM may exhibit distinct pathophysiological characteristics, including a comparatively greater impairment of steady-state beta-cell secretion than their obese counterparts [4]. In contrast to Western data, numerous prior Indian research indicate that a significant subset of T2DM patients are non-obese [5]. Previous research has also reported an "Asian Indian Phenotype," characterized by patients exhibiting elevated levels of visceral fat despite a lower body mass index (BMI) [6]. Cluster studies conducted worldwide have demonstrated that multiple clinical clusters exist among diabetic populations which have distinct phenotypical characteristics along with different pathophysiologic mechanisms predominating in each of them [7]. Therefore, in order to better understand the pathophysiology, it is imperative to examine the comparative beta-cell secretory function in T2DM patients across different BMI categories within the Indian population.

## Materials and methods

Study setting**

The study was done in the non-communicable disease (NCD) clinics of the family health centres (FHCs) in Kannur district, Kerala, India. The NCD clinics of the FHCs have a registry of all T2DM patients under their care.

Study design with study period**

This is a cross-sectional analytical study which was done over a period of two years from December 2021 to December 2023 after obtaining approval from the Institutional Ethics Committee of Government Medical College, Kannur (approval number: IEC No.93/2019/GMCK; date: 12/11/2020).

Study population

Patients who were registered in the NCD clinics of the selected FHCs in the geographical region were selected for the study based on the inclusion and exclusion criteria. Recent-onset T2DM patients diagnosed based on the American Diabetes Association 2010 criteria of age ≥30 years with a disease duration of ≤5 years were included in the study. Patients who were diagnosed as having chronic kidney disease, chronic liver disease, chronic infections, and malignancies were excluded. Also, those patients who were already on insulin therapy were also excluded. After the initial screening of patients satisfying the inclusion and exclusion criteria from the NCD records, they were classified based on their BMI into lean-weight (BMI <18.5 kg/m^2^), normal-weight (BMI ≥18.5 and <23 kg/m^2^), and overweight/obese (BMI ≥23 kg/m^2^) categories.

Patient selection and sample size calculation

The sample size for each of the subgroups was calculated based on the formula $\text{n}=2\times\left( \frac{\text{Z&alpha;}}{2}+\text{Z&beta;} \right)^{2}\times\frac{\text{\sigma}^{2}}{\text{d}^{2}}$. The $\frac{\text{Z&alpha;}}{2}$ value for a 99% confidence level (2.576) and the Zβ value for an 80% power (0.84) were used. Values from the reference study of Bautista et al. which compared the homeostasis model assessment 2-measure of insulin resistance (HOMA-IR) between lean-weight and overweight/obese diabetic patients were used for sample size calculation [8]. Sampling for the initial screening population was done in a multi-stage sampling, where initially nine FHCs were selected from the total 15 FHCs in the geographical area using the probability proportionate to size method. To account for this, a design effect of 2 was considered in the sample size calculation assuming the intra-cluster correlation of 0.01. After considering all these, a sample size of 35 per subgroup was obtained which was rounded off to 40 patients per subgroup. Forty patients were then selected from each of the body weight categories (a total of 120) from the initial screening group using simple random sampling for further study.

Data collection

Baseline data were collected from all 120 patients using a prevalidated proforma consisting of their personal details, past medical histories, and anthropometric measurements (namely, body weight, height, waist circumference (WC), and hip circumference (HC)). Detailed documentation was carried out for any oral hypoglycemic agents (OHAs) the patients were already using, including the specific type, the duration of use for each drug, and the daily dosage. Body weight and height were measured using a standard digital weighing machine and stadiometer, respectively. Other measurements were done by standard measuring tape. For the purpose of analysis, abdominal obesity was defined as a WC ≥90 cm in males and ≥80 cm in females based on the South Asian cut-off in International Diabetes Federation (IDF) guidelines. Following baseline data collection, fasting blood samples were obtained from all study subjects after ensuring eight hours of overnight fasting. Patients who were already on OHAs were allowed to continue the medications till the previous night due to ethical concerns. The fasting blood samples collected were centrifuged, and serum samples were transported to the tertiary care centre immediately for the estimation of fasting blood sugar (FBS) and fasting insulin (FI) levels. HOMA2 estimates for beta-cell secretion (HOMA2-%B) and insulin resistance (HOMA2-IR) were calculated using the HOMA2 calculator using FBS values and FI values measured from a single fasting serum sample (coupled) drawn after a minimum of eight hours of overnight fasting. The updated HOMA2 calculator (version 2.2.4) was downloaded from the University of Oxford, Center for Diabetes, Endocrinology and Metabolism, Diabetes Trial Unit (https://www.rdm.ox.ac.uk/about/our-clinical-facilities-and-mrc-units/DTU/software/homa/download), after necessary permission.

Data collection of the study was initiated after obtaining approval from the institutional ethics committee. Informed written consent was obtained from all patients before obtaining clinical data and a blood sample. The research work was done in accordance with the Declaration of Helsinki.

Statistical analysis

Data were compiled using LibreOffice Calc Version 6.4.7.2 (The Document Foundation (TDF), Berlin, Germany) and analyzed using RStudio Version 2023.06.1+524 (Posit Software, Boston, MA, USA). Descriptive data were represented using frequency, percentage, mean, and standard deviation. Normality of continuous variables was assessed using the Shapiro-Wilk test and normal QQ plotting. For comparing between different groups, appropriate tests like the Wilcoxon rank sum test, analysis of variance (ANOVA), and Kruskal-Wallis test were used. The Pearson correlation test or Spearman rank correlation test was employed to evaluate the correlation between various continuous variables and outcome variables based on the distribution's normality. The strength of the association between independent variables that exhibited a significant correlation with outcome variables, namely, HOMA2-IR and HOMA2-%B, was investigated using linear regression following the logarithmic transformation of non-normal variables. All statistical tests were two-tailed, with p≤0.01 as the cut-off for statistical significance in alignment with a 99% confidence level.

## Results

Out of the total 120 patients studied, 52.5% were females and 47.5% were males. The mean age of the study population was 59.93±9.19 years, and the mean duration of diabetes was 3.29±1.68 years. Out of the 40 lean-weight patients, only 37.5% had abdominal obesity, whereas the proportion of patients with abdominal obesity was higher in normal-weight (67.5%) and overweight/obese individuals (100%). Out of the continuous variables recorded, only age and systolic and diastolic blood pressures had a normal distribution. Other variables like duration of diabetes, HC and WC, FBS, FI, and HOMA2 outcome variables did not have a normal distribution.

All the variables were compared between the three body weight groups using appropriate statistical techniques depending on normality. Table 1 shows the comparative data of the three BMI category patients in our study group.

**Table 1:** Comparison of variables between various BMI categories The table compares various continuous variables between patients in the three BMI categories, namely, lean weight, normal weight, and overweight. Continuous variables are expressed in mean±standard deviation. A p-value of <0.01 was taken as a cut-off for statistical significance. BP: blood pressure; FBS: fasting blood sugar, HOMA2-%B: homeostasis model assessment 2-measure of beta-cell secretion; HOMA2-IR: homeostasis model assessment 2-measure of insulin resistance

Variable	Lean weight (40)	Normal weight (40)	Overweight (40)	P-value
Age	61.20±9.26	61.20±9.75	57.30±8.14	0.09
Duration of diabetes	3.34±1.81	3.47±1.61	3.06±1.62	0.46
Waist circumference	80.83±5.29	87.90±5.96	98.70±7.37	<0.001
Hip circumference	83.00±3.84	88.80±4.44	98.50±6.33	<0.001
Systolic BP	134.00±17.20	136.00±16.40	134.00±20.20	0.82
Diastolic BP	76.30±9.88	76.20±10.10	80.10±9.99	0.15
FBS	159.46±62.06	162.00±59.90	145.00±35.40	0.51
Fasting insulin	4.63±3.57	4.87±3.59	8.94±4.70	<0.001
HOMA2-%B	27.38±22.18	30.00±24.70	46.90±26.30	<0.001
HOMA2-IR	0.72±0.55	0.73±0.56	1.28±0.65	<0.001

**Figure 1:**
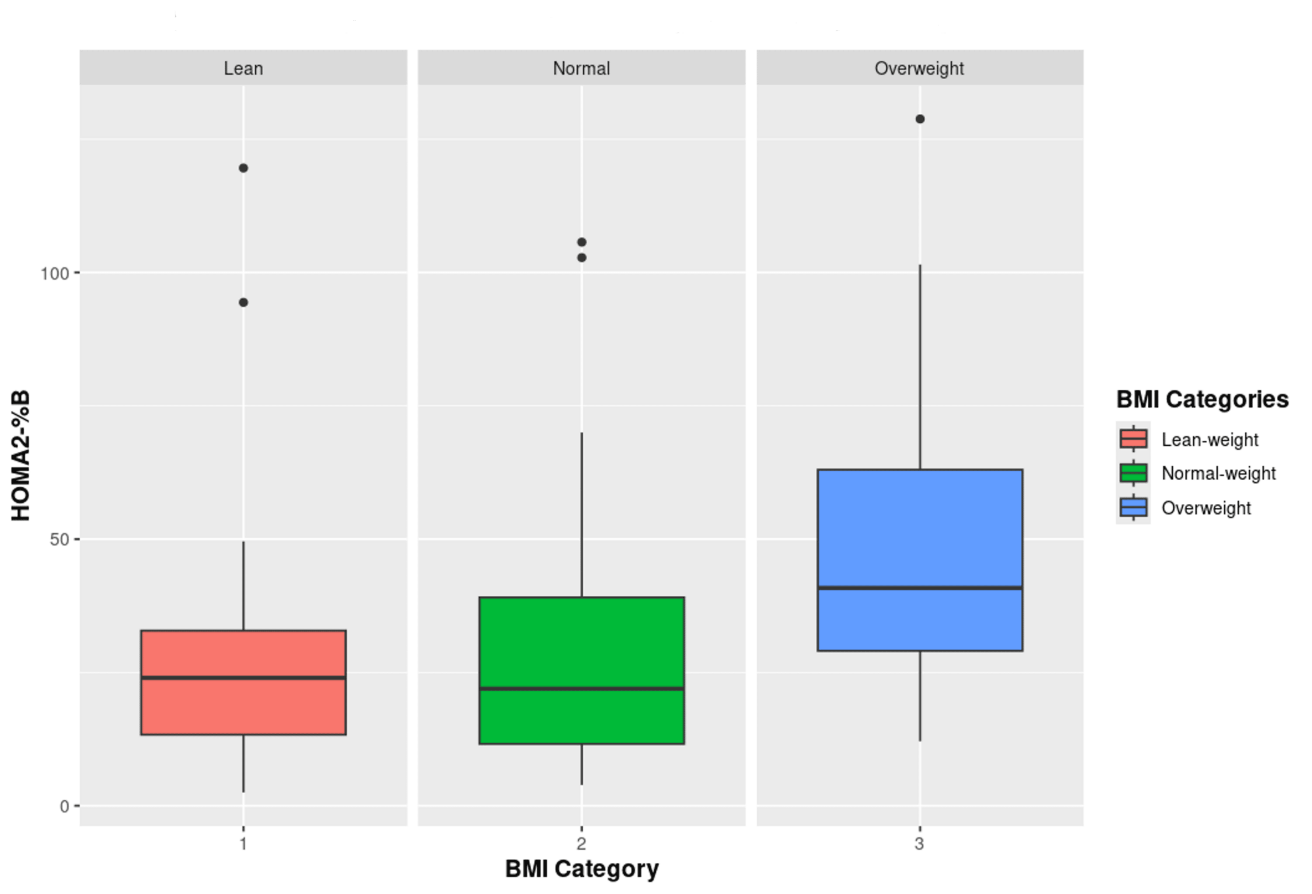
Comparison of beta-cell secretion among patients of BMI categories Comparison of HOMA2-%B between lean-weight, normal-weight, and overweight diabetic patients (p<0.001). HOMA2-%B: homeostasis model assessment 2-measure of beta-cell secretion; BMI: body mass index

**Figure 2:**
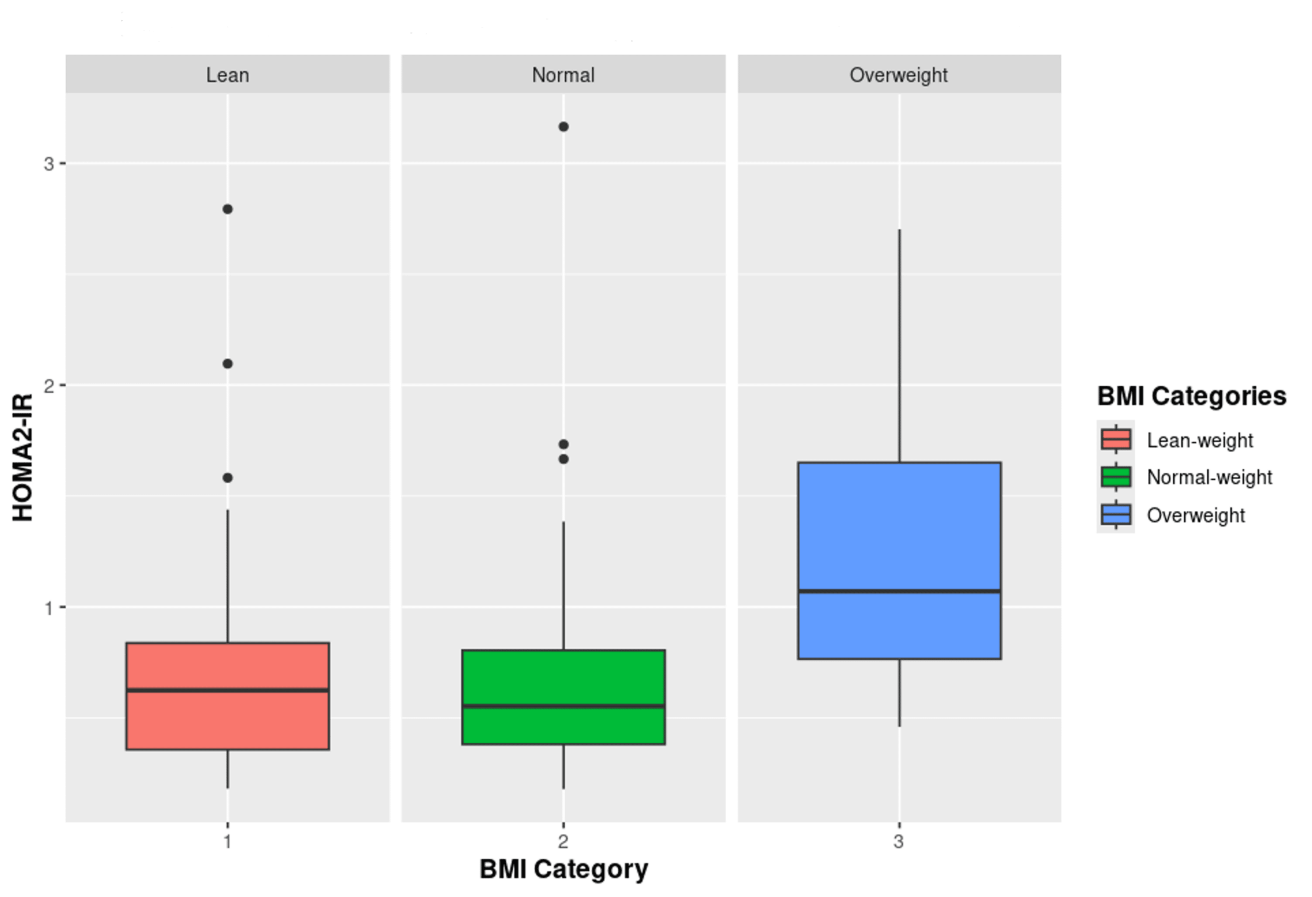
Comparison of insulin resistance among patients of BMI categories Comparison of HOMA2-IR between lean-weight, normal-weight, and overweight diabetic patients (p<0.001). HOMA2-IR: homeostasis model assessment 2-measure of insulin resistance; BMI: body mass index

Only WC, HC, FI, and HOMA2 values had statistically significant differences between the three BMI categories. Post hoc analysis further shows that out of these variables, only WC and HC had a significant difference between all the three groups. Others lacked a significant difference between the lean-weight and normal-weight groups, but each of them varied significantly from the overweight group (Figure 1 and Figure 2).

When beta-cell secretion and insulin resistance were compared among patients based on the presence of abdominal obesity, it was noted that patients with abdominal obesity had statistically significant higher values for both HOMA2-IR and HOMA2-%B compared to those who do not have (Table 2).

**Table 2:** Comparison of outcome variables based on abdominal obesity The table compares the values of outcome variables between patients based on their status of abdominal obesity. The variables are expressed in mean±standard deviation. A p-value of <0.01 was taken as a cut-off for statistical significance. HOMA2-%B: homeostasis model assessment 2-measure of beta-cell secretion; HOMA2-IR: homeostasis model assessment 2-measure of insulin resistance

Abdominal obesity	Absent	Present	P-value
HOMA2-IR	0.68±0.54	1.02±0.65	<0.001
HOMA2-%B	24.16±18.52	39.68±27.19	<0.001

Comparison of HOMA2-%B and HOMA2-IR values was done between patients who are already on OHA and those who are not taking OHA using the Wilcoxon rank sum test. No statistically significant difference was noted between HOMA2-%B (W=620; p=0.809) and HOMA2-IR (W=767; p=0.299) values between patients based on their OHA intake status.

Multiple regression analysis was done to predict HOMA2-%B and HOMA2-IR from clinical variables of patients including age, gender, WC, BMI, and OHA intake status. The model for the prediction of HOMA2-%B using these variables was found to be statistically significant (F(5, 114)=5.368; p<0.001; R2=0.155). For every 10% rise in BMI, HOMA2-%B is found to increase by 16.04%. Also, with every increasing year of age of presentation, the patient is predicted to have 1.71% higher value of HOMA2-%B. Female patients have 41.55% higher HOMA2-%B level compared to their male counterparts. Similarly, the model for the prediction of HOMA2-IR using the same variables also was statistically significant (F(5, 114)=9.644; p<0.001; R2=0.266). Here, for every 10% increase in BMI, the HOMA2-IR rises by 19.34%. Females are predicted to have 31.78% higher HOMA2-IR values than males. Similarly, those patients who are currently on one or more OHA have 59.96% lower HOMA2-IR values than patients who are not on any OHA.

## Discussion

Relative beta-cell secretory defect is an important pathophysiological aspect in the development of T2DM. In our study, we intended to investigate the variability in beta-cell secretion across T2DM patients of different BMI categories in the South Indian population. Even though abdominal obesity was present in all categories of diabetic patients in our study, it was lesser in proportion in patients who were having lean-weight compared to normal-weight or overweight patients. So both abdominal obesity and general obesity go hand in hand in T2DM patients in our region. Similar studies done in various parts of the globe have also concluded that both general and abdominal adiposity are independent risk factors in the development of T2DM in individuals [9,10]. Indian studies have especially stressed upon the role of abdominal obesity in the incidence of T2DM in our population [11].

Our study found that among the three BMI categories, overweight/obese diabetic patients had significantly higher values of beta-cell secretion (HOMA2-%B) and insulin resistance (HOMA2-IR) than lean-weight and normal-weight diabetic patients. In contrast, there was no statistically significant difference between the latter two groups on these measures. Fan et al. found that among Chinese patients with young-onset diabetes, those of normal weight exhibited reduced beta-cell secretory function and preserved insulin sensitivity in comparison to their obese counterparts [4]. Other studies evaluating these parameters across various BMI categories in T2DM patients reached a similar conclusion. Lean diabetic patients exhibited lower beta-cell secretion than non-lean diabetic patients, with no significant difference in insulin resistance [8]. Obesity is known to increase insulin resistance among patients, and the comparative rise in beta-cell secretion in these individuals is expected as a response to it. But the observation that diabetic patients who are not overweight/obese have lower beta-cell secretion with better insulin sensitivity needs further inquiry. These patients may represent distinct clinical clusters of T2DM having underlying genetic variability contributing to their lower beta-cell secretion as has been suggested in recent cluster studies [7].

The current study also concluded that there is a significant difference between HOMA2-%B and HOMA2-IR values between diabetic patients based on the presence of abdominal obesity. Both these values were found to be significantly higher in patients who have abdominal obesity. Xu et al., in their study from China, observed that like overall obesity, abdominal obesity also had an independent causative role in T2DM and insulin resistance [12]. Gastaldelli et al. showed that visceral adiposity is a part of the insulin resistance phenotype and increased insulin secretion to a physiological challenge is largely a compensatory response to this rise in insulin resistance [13]. All these reports suggest that abdominal obesity is primarily linked to increased insulin resistance and an appropriate rise in beta-cell secretory response.

Other factors that seem to influence beta-cell secretion are the gender and age of the patient. Females seem to have higher HOMA2-%B values, hence possibly having better preserved beta-cell function compared to their male counterparts. They also have higher HOMA2-IR values than males. Almost 85.71% of our female patients were above the age group of 50 years, and this could explain the rise in insulin resistance in them as suggested in previous studies which investigated the gender disparity of diabetic patients. The increased adiposity and change in fat distribution in the postmenopausal period have been suggested as the cause for this observation by the authors [14]. Since the current study only included patients with new-onset diabetes, the increase in beta-cell secretion with patient age observed in it may be interpreted as suggesting that patients with diabetes who develop the disease later in life have more preserved beta-cell function than those who do so earlier. But insulin resistance (HOMA2-IR) did not have a significant variation with respect to rising age. Even though many of the previous studies failed to demonstrate any significant effect of age on glucose homeostasis, some studies noted a decrease in insulin sensitivity with age which was attributed to the increased adiposity that comes about with increasing age [15,16].

Limitations

During the study, the subjects were allowed to take OHA till the previous night of blood sample collection due to ethical reasons. This might have affected the HOMA2 variables of individual patients as OHAs will interfere with the beta-cell secretion and insulin resistance. This limitation was mitigated by collecting detailed data about the OHA intake of each patient. We then included the factor of OHA intake during our regression analysis and assessed its effect on both beta-cell secretion and insulin resistance.

## Conclusions

The current study hence throws light on the fact that lean-/normal-weight diabetic patients have comparatively lower beta-cell secretion with better insulin sensitivity compared to patients who are overweight/obese, whereas patients with abdominal obesity exhibited significantly higher insulin resistance, along with better preserved beta-cell function. Late-onset diabetic patients retain better beta-cell reserve compared to young-onset diabetic patients. Likewise, female gender seems to have higher insulin resistance with preserved beta-cell secretion compared to their male counterparts. This study provides significant insights into the relative measure of beta-cell activity and insulin resistance in T2DM patients, based on their diverse clinical characteristics, particularly body habitus, which will have crucial implications for the clinical management of diabetic patients in day-to-day practice. Policy implications of these findings are important from a public health perspective since they point towards the requirement of tailor-made treatment selection for each patient based on their clinical and anthropometric characteristics.
